# *PPARG* Hypermethylation as the First Epigenetic Modification in Newly Onset Insulin Resistance in Human Adipocytes

**DOI:** 10.3390/genes12060889

**Published:** 2021-06-09

**Authors:** Małgorzata Małodobra-Mazur, Aneta Cierzniak, Krzysztof Kaliszewski, Tadeusz Dobosz

**Affiliations:** 1 Department of Forensic Medicine, Wroclaw Medical University, Sklodowskiej-Curie 52, 50-369 Wroclaw, Poland; aneta.cierzniak@student.umed.wroc.pl (A.C.); tadeusz.dobosz@umed.wroc.pl (T.D.); 2 Department of Molecular Techniques, Wroclaw Medical University, Sklodowskiej-Curie 52, 50-369 Wroclaw, Poland; 3 Department of General, Minimally Invasive and Endocrine Surgery, Wroclaw Medical University, Borowska 213, 50-556 Wroclaw, Poland; krzysztof.kaliszewski@umed.wroc.pl

**Keywords:** insulin resistance, *PPARG*, DNA methylation, *DNMT1*, SAT, VAT

## Abstract

Insulin acts by binding with a specific receptor called an insulin receptor (INSR), ending up with glucose transporter activation and glucose uptake. Insulin resistance (IR) is a state when the physiological amount of insulin is not sufficient to evoke proper action, i.e., glucose uptake. Epigenetic modifications associated with obesity and IR are some of the main mechanisms leading to IR pathogenesis. The mesenchymal stem cells of adipose tissue (subcutaneous (SAT) and visceral (VAT)) were collected during abdominal surgery. IR was induced ex vivo by palmitic acid. DNA methylation was determined at a global and site-specific level. We found higher global DNA methylation in IR adipocytes after 72 h following IR induction. Furthermore, numerous genes regulating insulin action (*PPARG*, *SLC2A4*, *ADIPOQ*) were hypermethylated in IR adipocytes; the earliest changes in site-specific DNA methylation have been detected for *PPARG*. Epigenetic changes appear to be mediated through DNMT1. DNA methylation is an important component of IR pathogenesis; the *PPARG* and its epigenetic modification appear to be the very first epigenetic modification in newly onset IR and are probably of the greatest importance.

## 1. Introduction

Insulin acts by binding with a specific receptor called an insulin receptor (INSR) that, while activated, undergoes autophosphorylation and further phosphorylation of downstream kinases, ending up with glucose transporter activation, its translocation to the cell membrane, and glucose uptake. When the process of signal transduction is aborted, insulin resistance develops. Insulin resistance (IR) is defined as a state when the physiological amount of insulin does not evoke proper action, which is glucose utilization by peripheral tissues [1,2]. IR is a serious epidemiologic problem, mainly in highly industrialized countries, affecting millions of people each year [3,4].

The proper insulin response depends on many mechanisms and signaling pathways in the cell. By far the most important element of the insulin respond regulation is the nuclear receptor family of PPARs, especially PPARG [5]. It plays a key role in adipogenesis [6] and the regulation of lipid metabolism [7]. Additionally, PPARG is crucial for glucose metabolism regulation; indeed, drugs acting as PPARG agonists significantly abolish peripheral insulin resistance [8].

The precise mechanism of insulin resistance pathogenesis is not fully understood. The main observed disorder in insulin-resistant cells is the disturbance of lipid metabolism in the form of an increase in free fatty acids. Additionally, there is a defect in the mitochondria relying on the reduction of oxidative phosphorylation [2].

Factors that increase the risk of IR include a sedentary lifestyle, inappropriate diet, and an excess caloric intake [9,10]. In patients with impaired insulin sensitivity, numerous genes involved in insulin action and/or insulin sensitivity regulation are expressed differently [11]. Epigenetics is one of the mechanisms regulating gene expression [12,13].

Epigenetics refers to gene expression regulation arising from chromatin marks, including DNA methylation. DNA methylation induces chromatin remodeling, affecting gene expression. Generally, hypermethylation of DNA is linked with transcriptional inactivity, whilst hypomethylation of DNA is linked with transcription activation [14].

Changes in DNA methylation associated with IR have been shown in numerous studies, both globally and concerning particular CpG sites [11,15,16,17,18]. The global DNA methylation measured in peripheral blood leukocytes was associated with HOMA-IR in monozygotic twins [16]. Furthermore, shreds of evidence provided by Grundberg et al. concerning the analysis of global DNA methylation patterns in adipose tissue of monozygotic and dizygotic twins showed a correlation between DNA methylation of particular genes associated with metabolic diseases such as glucose-metabolism disorders, IR, and type 2 diabetes [17]. Similarly, Hidalgo et al. showed that particular CpGs methylation patterns were associated with insulin and IR based on HOMA-IR [18]. Numerous studies support the role of epigenetic factors in the development of IR and indicate that DNA methylation may play a role in IR and diabetes pathogenesis [11,15,16,17,18].

In our previous research, we have shown dysregulation in the expression of numerous genes in adipose tissue of insulin-resistant patients that arises from changes in DNA methylation [11]. In the present paper, we were trying to assess the timeline of changes in DNA methylation that underly insulin resistance. Furthermore, we compared the changes in DNA methylation in adipocytes collected from subcutaneous (SAT) and visceral (VAT) tissue after the induction of insulin resistance.

## 2. Materials and Methods

The study protocol was approved by the Ethics Committee Board of Wroclaw Medical University, Approval No. KB-124/2017.

### 2.1. Human Preadipocyte Collection and Differentiation

Human mesenchymal stem cells (MSC) of white adipose tissue were extracted from SAT and VAT tissue collected from three patients during routine surgery. Enrolled patients had normal BMI (23 ± 1.4 kg/m^2^), insulin (8.0 ± 1.4 µU/mL), glucose (94 ± 4 mg/dL), and lipids levels (TG 76 ± 11 mg/dL; HDL 60 ± 17 mg/dL; LDL 116 ± 29 mg/dL). Furthermore, patients declared that no type 2 diabetes had been diagnosed in close relatives. The mean age of patients was 44 ± 5 years. All enrolled patients were men.

The collected tissues were placed in PBS (IDT) supplemented with a mix of protease inhibitors (PI, Sigma-Aldrich, St. Louis, MO, USA) and transported to the lab facility. Next, the tissues were dissected, visible blood vessels were removed, and tissue digested with collagenase (1 mg/mL medium) supplemented with BSA (10 mg/mL medium, Sigma-Aldrich) until complete digestion. After digestion, cells were centrifuged for 5 min at 2000 rpm; the supernatant was discarded, and cells were washed twice with ice-cold PBS. After the final centrifugation, the cells were suspended in DMEM/F12 supplemented with 10% FCS (Sigma-Aldrich) and antibiotics (50 U/mL of penicillin and 50 µg/mL of streptomycin, Corning, New York, NY, USA).

The culture medium was replaced every second day until confluent. The differentiation cocktail contained DMEM/F12 (50:50) supplemented with 10% FCS, penicillin (50 U/mL), streptomycin (50 µg/mL), IBMX (115 µg/mL), dexamethasone (390 ng/mL), insulin (10 µg/mL), pioglitazone (0.1 µg/mL), and human transferrin (10 µg/mL) (all purchased in Sigma-Aldrich). After three days, the differentiation cocktail was replaced with medium containing DMEM/F12 (50:50) supplemented with 10% FCS, penicillin (50 U/mL), streptomycin (50 µg/mL), insulin (10 µg/mL), pioglitazone (0.1 µg/mL), and human transferrin (10 µg/mL) for anadditional three days. At the end of adipogenesis, cells were cultured with a medium containing DMEM/F12 (50:50) and 10% FCS and antibiotics. After the following three to four days, the cells were fully mature adipocytes. All experiments were performed in triplicate. 

### 2.2. T3-L1 Cell Line Culture and Differentiation

Next, 3T3-L1 were differentiated after achieving 100% confluence. The differentiation medium contained DMEM (Dulbecco’s Modified Eagle Medium, Corning), 10% fetal bovine serum (FBS, Corning), antibiotics (penicillin, 50 U/mL; streptomycin, 50 µg/mL, Corning), 3-isobutyl-1-methylxanthine (115 µg/mL, Sigma-Aldrich), dexamethasone (390 ng/mL, Sigma-Aldrich), and insulin (10 µg/mL, Sigma-Aldrich). After three days, the medium was changed to DMEM with antibiotics, 10% FBS and insulin (10 µg/mL). After three more days, the medium was changed to DMEM with antibiotics, 10% FBS, and further cultured for an additional two days to achieve a fully mature phenotype. All experiments were performed in triplicate.

### 2.3. Insulin Resistance Induction

Insulin resistance was induced in the experimental mature adipocytes by 0.5 mM palmitic acid (16:0). The IR was induced for 48 h and 72 h. After each time point, a glucose uptake test was completed using Glo-Glucose Uptake (Promega, Madison, WI, USA) to assess the glucose uptake rate of the mature adipocytes. Glucose uptake measurements were performed according to manufacturer’s protocol.

### 2.4. Genetic Material Extraction

DNA was extracted using phenol:chloroform:isoamyl alcohol reagent (25/24/1, *v/v/v*, (BioShip, Waltham, MA, USA). DNA was suspended in nuclease-free water and stored at −20 °C.

RNA was extracted using the TriReagent (Sigma-Aldrich) reagent followed by isopropanol (Sigma-Aldrich) precipitation. After drying, the RNA was suspended in nuclease-free water and stored at −80 °C.

### 2.5. Gene Expression

The RNA (200 ng) was transcribed into cDNA using the high capacity reverse transcription kit (ThermoFisher Scientific, Waltham, MA, USA). Gene expression was analyzed in real-time PCR using the Fast SYBR Green Master Mix (ThermoFisher Scientific). Primers were designed manually and the efficiency of primers was checked by a standard curve. The sequence of primers was published previously [11,19]. Normalization was executed on the housekeeping gene (β-actin) and calculated according to the ΔΔCt algorithm.

### 2.6. Global and Site-Specific DNA Methylation

DNA methylation was analyzed using the MagMeDIP qPCR Kit (Diagenode, Denville, NJ, USA) according to protocol. After precipitation and DNA extraction, the DNA concentration was measured using Pico488 dsDNA quantification reagent (Lumiprobe, Hannover, Germany). The standard curve was prepared using human DNA quantified by the Quantifiler™ Duo DNA Quantification Kit (ThermoFisher Scientific). Global DNA methylation was calculated as the percentage of DNA immunoprecipitated using C^me^ antibodies (Diagenode) to the input amount of DNA. The site-specific DNA methylation was analyzed in real-time PCR using the Fast SYBR Green Master Mix (ThermoFisher Scientific). The primer sequences are presented in Table 1. The calculation of percent of input was performed according to the manufacturer protocol:

[% recovery = 2^(CtIN−3.32)–CtIP)^ ∗ 100

CtIN—Ct value of 10% input; CtIP—Ct value of immunoprecipitated samples].

### 2.7. Statistical Analysis

Statistical analysis was performed using Statistica13.1 (StatSoft). For analysis differences between groups, a *t*-test was used. To assess the correlation between numerical characteristics, a correlation of coefficient was used. The normality of the variable distribution was checked using the W Shapiro–Wilkes test. Gene expression was calculated using the ΔΔCt algorithm. The % of DNA methylation was calculated according to provided formula. Statistical significance was set at *p* < 0.05.

## 3. Results

### 3.1. Global DNA Methylation in Insulin-Resistant Adipocytes

**Figure 1:**
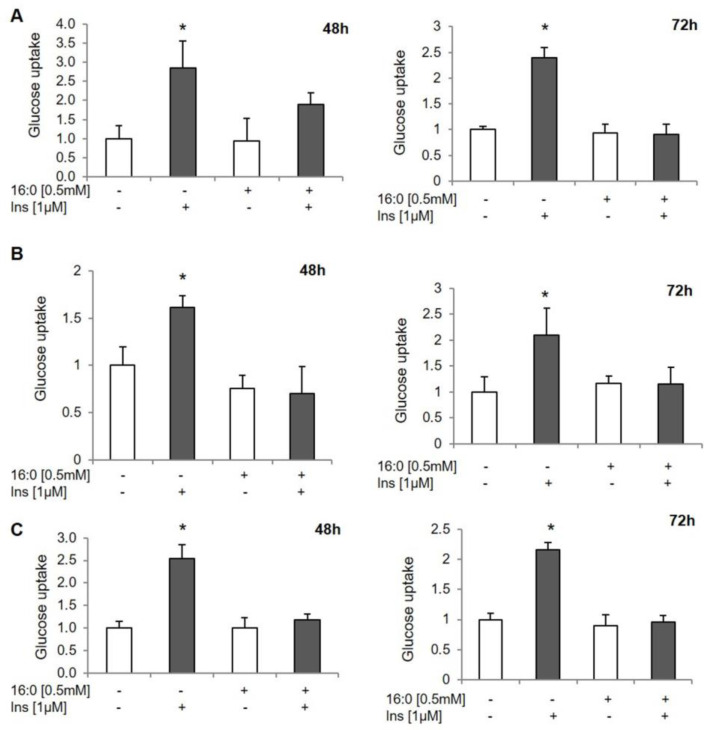
The assessment of insulin resistance in experimental adipocytes. The glucose uptake measurements in control and insulin-resistant adipocytes treated with 0.5 mM palmitic acid (16:0) for 48 h and 72 h: (**A**) 3T3-L1 adipocytes, (**B**) human SAT-derived adipocytes, (**C**) human adipocytes from VAT, INS−—basal glucose uptake, INS+—insulin-stimulated glucose uptake, * *p* < 0.05.

Previously, we showed an altered global DNA methylation profile in the adipose tissue biopsies of insulin-resistant patients that was positively correlated with an insulin-resistant state [11]. To confirm the relationship between IR and epigenetic regulation, and also to evaluate the timeline of changes in DNA methylation, we induced IR in 3T3-L1 mature adipocytes and human adipocytes collected from VAT and SAT. The IR was induced by 0.5 mM of palmitic acid (16:0) for 48 h and 72 h. The glucose uptake test confirmed that the cells treated with palmitic acid were resistant to insulin. In cells treated with palmitic acid (16:0), the insulin-stimulated glucose uptake was the same as the basal glucose uptake in cells treated with palmitic acid. In control cells with proper insulin sensitivity, insulin-stimulated glucose uptake increased about 2–3-fold compared to basal glucose uptake in all experimental cells (Figure 1).

**Figure 2:**
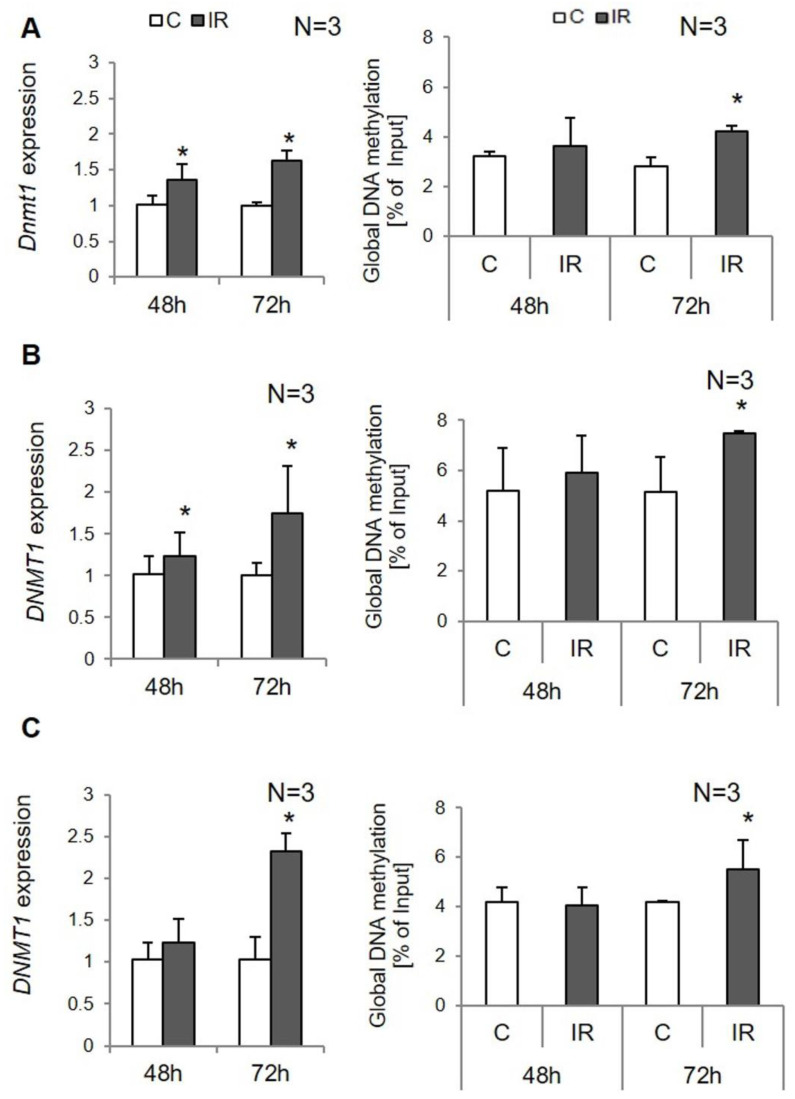
The *DNMT1* expression and global DNA methylation in experimental cells following 48 h and 72 h after insulin resistance induction. Controls (C) and insulin-resistant cells (IR): (**A**) 3T3-L1 adipocytes, (**B**) human SAT-derived adipocytes, (**C**) human VAT-derived adipocytes; gene expression is normalized to β-actin gene, * *p* < 0.05.

The global DNA methylation in insulin-resistant cells was increased in all types of examined cells. However, changes were significant only after 72 h of IR induction. In 3T3-L1 adipocytes after 72 h of IR induction, DNA methylation increased significantly in IR cells compared to controls (*p* = 0.041; see Figure 2).

Similarly, in human adipocytes, global DNA methylation was increased both in SAT- and VAT-derived adipocytes with IR after 72 h of IR induction (*p* = 0.035 and *p* = 0.025, respectively; see Figure 2).

Regarding the expression of epigenetic regulatory genes, we found an increased *DNMT1* expression in IR adipocytes in all experimental cells (Figure 2). We detected overexpression of *DNMT1* in 3T3-L1 adipocytes with IR compared to controls; there was a 1.5-fold increase after 48 h and about 1.8-fold after 72 h of IR induction (48 h: *p* = 0.001; 72 h: *p* < 0.000;). Similarly, in insulin-resistant human adipocytes, we observed overexpression of the *DNMT1* gene compared to controls. The increase in *DNMT1* expression in SAT-derived adipocytes was similar as observed in 3T3-L1 (48 h: *p* = 0.040; 72 h: *p* = 0.005). In VAT-derived adipocytes, the *DNMT1* was overexpressed only after 72 h of IR induction for about 2.5-fold (48 h: *p* = 0.463; 72 h: *p* = 0.006; Figure 2). No other methyltransferases were shown to be differently expressed.

### 3.2. Site-Specific DNA Methylation in Insulin-Resistant 3T3-L1 Adipocytes

**Figure 3:**
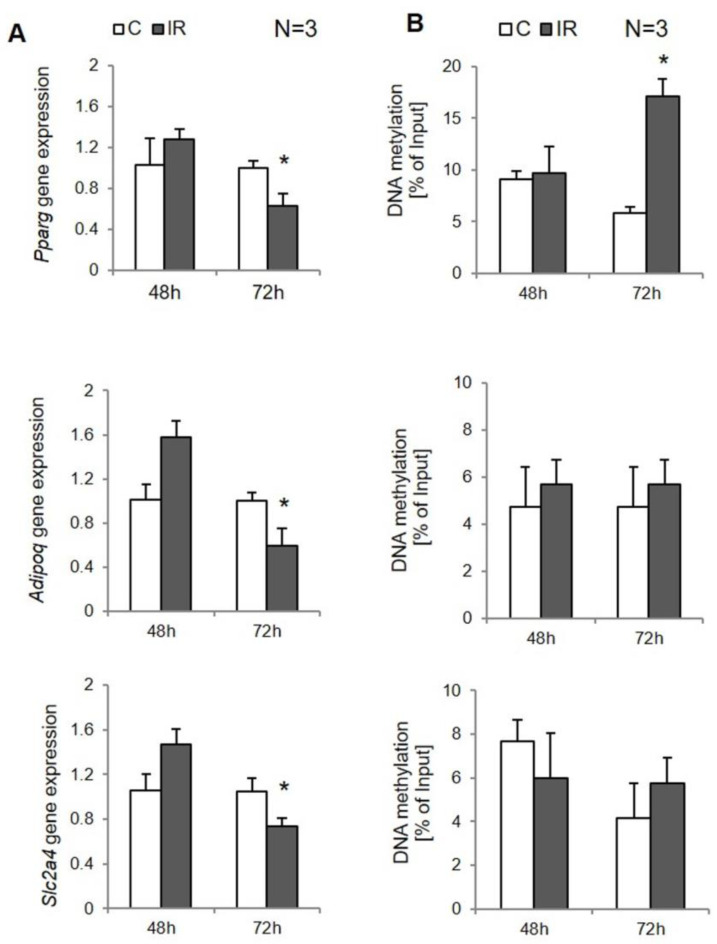
The insulin-sensitizing genes’ expression and their site-specific DNA methylation in 3T3-L1 adipocytes. Controls (C) and induced insulin resistance (IR): (**A**) gene expression (normalized to β-actin gene), (**B**) site-specific DNA methylation (% of input), * *p* < 0.05.

First of all, the expression rate of numerous genes crucial for insulin signaling was measured in control cells and IR adipocytes. In 3T3-L1 cells after 48 h of IR induction, any significant changes in the expression rate of insulin pathway genes or lipid metabolism genes, or genes regulating insulin sensitivity were reported. However, after 72 h of IR induction, downregulation of the following genes in IR cells compared to controls was observed: *Slc2a4* (*p* = 0.002), *Adipoq* (*p* < 0.000), *Pparg* (*p* < 0.000; Figure 3A).

DNA methylation at the protomer sites of the analyzed genes did not differ between experimental cells, except the *Pparg* gene, where the methylation rate was twice as high in IR cells compared to controls after 72 h of IR induction (*p* < 0.000; Figure 3B). At the same time, in experimental adipocytes 72 h after induction of insulin resistance, a negative correlation was observed between methylation of the *PRRAG* promoter and its expression (R = −0.78, *p* = 0.038). No other correlations were noticed.

### 3.3. Site-Specific DNA Methylation in Insulin-Resistant Human Adipocytes

**Figure 4:**
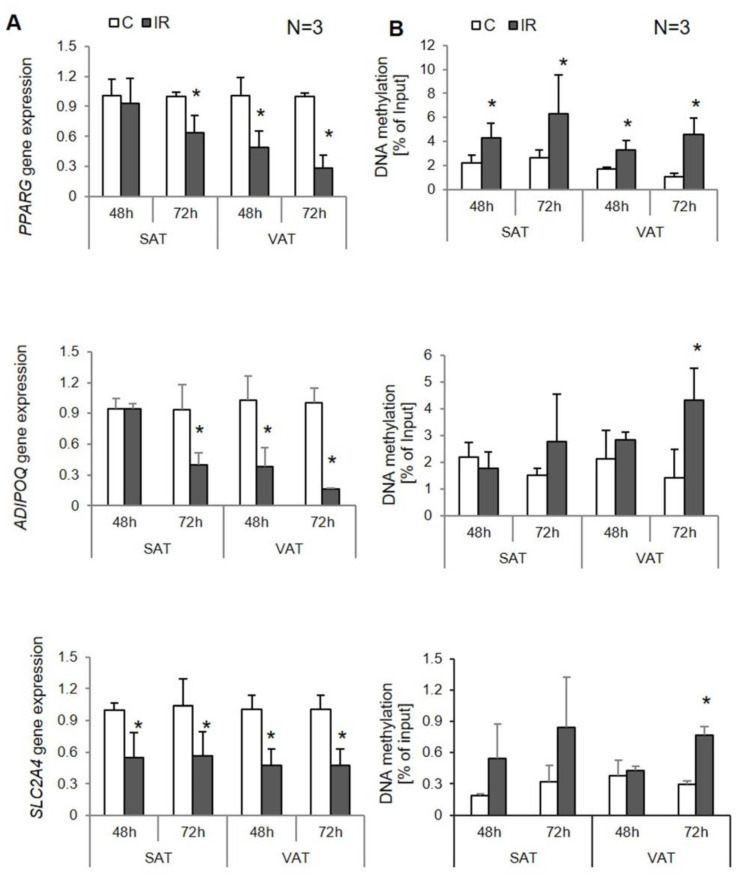
The insulin-sensitizing genes expression and their site-specific DNA methylation in human adipocytes (SAT and VAT). Controls (C) and induced insulin resistance (IR): (**A**) gene expression (normalized to β-actin gene), (**B**) site-specific DNA methylation (% of input), * *p* < 0.05.

In insulin-resistant human adipocytes collected from SAT, *ADIPOQ* and *PPARG* expressions decreased around two-fold after 72 h of IR induction compared to control cells (*p* = 0.001 and *p* = 0.002, respectively; Figure 4A). Additionally, the *SLC2A4* gene was downregulated in IR adipocytes at both time points: after 4 8 h and 72 h of IR induction (*p* = 0.044 and *p* = 0.006, respectively).

Contrary to SAT-derived adipocytes, in VAT-derived adipocytes, changes in expression of analyzed genes were detected at both time points: after 48 h and 72 h. The expression of the *SLC2A4* gene was decreased by about 50% in insulin-resistant adipocytes compared to controls (48 h: *p* = 0.015, 72 h: *p* = 0.001). The expression of the *ADIPOQ* gene decreased about 50% in IR cell after 48 h (*p* = 0.013) and about 80% after 72 h (*p* = 0.012). The *PPARG* gene was downregulated by about 50% after 48 h (*p* = 0.002) and 70% after 72 h (*p* = 0.004; Figure 4A) in IR adipocytes compared to controls. Additionally, the *INSR* gene was also downregulated after 48 h (*p* = 0.046) and 72 h (*p* = 0.002) of IR induction in VAT-derived adipocytes.

In insulin-resistant SAT-derived adipocytes, the methylation of the *SLC2A4* and *ADIPOQ* promoters were increased at both time points without being statistically significant. However, a strong negative correlation between *SLC2A4* expression and promoter methylation was observed in experimental adipocytes at the second time point (R = −0.95, *p* = 0.003). The methylation of the *PPARG* promoter was higher by about two times after 48 h (*p* = 0.006) and three times after 72 h (*p* = 0.024) of insulin resistance induction compared to adipocytes with proper insulin sensitivity; this correlated with the expression of this gene in experimental adipocytes. However, the correlation was only noticed after 72 h following insulin resistance induction (R = −0.77, *p* = 0.024). There was only a slight negative correlation reported after 28 h of IR induction.

In insulin-resistant VAT-derived adipocytes, the hypermethylation of *SLC2A4* gene and *ADIPOQ* gene promoters were detected; however, this only occurred after 72 h of IR induction (*ADIPOQ*, *p* = 0.049; *SLC2A4*, *p* = 0.018; Figure 4B), and the methylation level was three times higher in insulin-resistant adipocytes compared to controls. Furthermore, similar to SAT-derived adipocytes, the methylation of *SLC2A4* negatively correlated with gene expression (R = −0.97, *p* < 0.000). After 48 h, there were neither differences in promoter methylation between experimental and control cells nor was there a correlation with the expression. On the other hand, the *PPARG* promoter was hypermethylated at both time points, which increased as insulin resistance developed from a two-fold increase after 48 h (*p* = 0.032) to a triple increase after 72 h (*p* = 0.010). Despite the increased methylation rate at both time points, the negative correlation with gene expression was reported after only 72 h of IR induction (R = −83, *p* = 0.011). After 48 h, only a slight correlation was reported (R = −0.44, *p* = 0.138).

## 4. Discussion

DNA methylation is one of the epigenetic regulatory mechanisms that regulate the organism’s adaptation to various environmental factors. DNA methylation has been implemented in the pathogenesis of numerous diseases including cancers, neurodegeneration diseases, and metabolic disorders [11,20,21].

In this paper, we showed the timeline of changes in DNA methylation in adipocytes at the very early stages of newly developed insulin resistance. We demonstrated that DNA methylation is an essential component of IR pathogenesis in adipose tissue. The IR was induced in experimental cells: 3T3-L1 adipocytes and human adipocytes collected from SAT and VAT by palmitic acid, which is one of the most common methods used by numerous researchers [22,23]. We successfully induced IR both in 3T3-L1 and human adipocytes, which was confirmed by a glucose uptake assay. At both time points (48 h and 72 h), the cells were resistant to insulin.

Aberrant global DNA methylation and its links with IR have been reported by numerous authors [11,15,16,17]. In our previous research, we indicated the correlation between DNA methylation and obesity [11,15]. The previous study showed an increased global DNA methylation in both analyzed fat depots of IR patients and its positive correlation with the IR ratio (HOMA-IR), which is consistent with other research. Similar to our previous results that were performed mainly using adipose tissue biopsies, we have shown global DNA hypermethylation in experimental adipocytes with IR induced ex vivo. Analyzing the timeline, we observed that global DNA methylation was increased as soon as 72 h following IR induction, which suggests that changes in the epigenome occur at the early stages of the development of insulin resistance.

In our previous results, we found higher expression of *DNMT1* in adipose tissue of IR patients as the potential reason for DNA hypermethylation [11]. Similar implementation of *DNMT1* in IR pathogenesis has been observed by others, where an increased expression of *DNMT1* correlated with obesity and IR [24,25]. On the other hand, inhibition of its activity ameliorates obesity, inflammation, and IR in cell culture or animal models [24,26]. In the present study, we observed, similar to in vivo results, *DNMT1* overexpression in IR adipocytes, both in 3T3-L1 and human SAT-derived and VAT-derived adipocytes as soon as 48 h following IR induction. The obtained results prove the implementation of *DNMT1*, not other methyltransferases, in IR pathogenesis, which is similar to results observed in adipose tissue samples [11]. The obtained results suggest that *DNMT1* overexpression is one of the mechanisms that is developed in the cell at the very early stages of IR induction.

DNA methylation is certainly the most widely studied epigenetic modification, and the link between promoter methylation of insulin signaling genes has been confirmed by other researchers in numerous studies [11,15,24,27,28]. We also considered the site-specific epigenetic modification at the promoter site of primary insulin-related genes.

First, we analyzed the expression rate of numerous genes in experimental cells. Several genes were differently expressed in IR adipocytes, including *PPARG*, *SLC2A4*, and *ADIPOQ*, similar to the results that we observed in vivo [11] and similar to other studies [15,24,29]. However, there were slight differences in the expression of analyzed genes between SAT and VAT-derived adipocytes. In SAT-derived adipocytes, only the *SCL2A4* gene, encoding GLUT4, had been downregulated after 48 h of IR induction. This is contrast to VAT-derived adipocytes, which were characterized by lower expression of all mentioned genes, both after 48 h and 72 h of IR induction. We have recently shown that SAT and VAT fat depot differ significantly, especially according to the risk of metabolic disorder developments. Probably due to higher inflammatory markers in VAT, this type of tissue has been associated with a higher risk of metabolic disorder pathogenesis [19]. The 3T3-L1 adipocytes revealed expression similar to SAT adipocytes; the reduction was seen only at the second time point, which means that this type of cell is more similar to the SAT depot and does not correspond to the VAT depot.

PPARG is one of the most important transcription factors regulating insulin sensitivity. The reduction in *PPARG* expression in IR has been shown by numerous researchers. The same applies to the finding related to *PPARG* promoter hypermethylation [27,30], which is consistent with our findings. In the present study, we have shown that *PPARG* hypermethylation was the first epigenetic modification observed in adipocytes following insulin resistance induction as soon as 48 h after IR induction. Although the statistically significant negative correlation between *PPARG* expression and methylation was observed at the second time point, a trend of decreasing *PPARG* expression was observed, with increasing methylation of its promoter, as soon as 48 h after IR induction. The obtained results prove that changes in *PPARG* DNA methylation are the first component of IR development. What is more, the epigenetic changes and their effect on *PPARG* expression were observed in all experimental models, including both depots of human adipose tissue and experimental cells, which proves the critical role of *PPARG* in insulin signaling regulation by the epigenetic modification that have a place within this gene.

The latest results suggest that methylation of the *SLC2A4* gene might be used as a biomarker of IR [28]. Indeed, in our study, the *SLC2A4* gene was shown to be expressed at a lower rate in IR cells as one of the first genes being downregulated after just 48 h of IR induction in both types of adipocytes. Furthermore, as was mentioned above, dysregulation in *SLC2A4* expression/translocation is believed to be the first molecular change in IR pathogenesis. The present study also confirmed the higher rate of the *SLC2A4* promoter’s methylation in IR adipocytes, especially in VAT. However, despite a decreased *SLC2A4* expression in human adipocytes cultured in vitro after 48 h of IR induction, the methylation rate was found to be increased after only 72 h. Moreover, it was only at this time point that it was negatively correlated with gene expression. In the regulation of *SLC2A4* expression, other mechanisms might be involved. *SLC2A4* expression is regulated by MEF2 (myocyte enhancer factor) and other transcription factors, including *PPARG*.

The DNA hypermethylation of the *ADIPOQ* promoter has been previously linked with obesity and IR [24]. All findings of in vivo studies were confirmed in experimental cells. However, the DNA hypermethylation was significant in VAT-derived adipocytes only; in SAT-derived adipocytes, similar to 3T3-L1, the reported increase was not significant.

The presented work also has a few limitations that should be discussed. First of all, the amount of available adipose tissue from which MSC was obtained is relatively low; it also came solely from men. Second, IR was induced by one factor, palmitic acid. In fact, in vivo, many different factors, such as others nutrients, lack of exercise, and inflammation, contribute to the development of insulin resistance. However, in the authors’ opinion, obtaining similar results on three cell models may suggest that the epigenetic modifications, including DNA methylation, participate in the pathogenesis of insulin resistance.

## 5. Conclusions

In conclusion, DNA methylation is an important component of IR pathogenesis, and the changes occur at the very early stages of insulin resistance development. Changes in global DNA methylation were detected after 72 h of IR induction. On the other hand, the changes in particular CpG sites were detected just after 48 h of IR induction. Based on the obtained results, the *PPARG* and its promoter’s methylation appear to be the very first epigenetic modification in newly onset IR, and are probably of the greatest importance.

## Figures and Tables

**Table 1:** Primers sequences used for DNA methylation analysis.

Gene	Species	Sequence [3′→5′]		Amplicon [bp]	CpG *
*PPARG*	Human	F	CTGTTATGGGTGAAACTCTGG	58	4
		R	GTGAAGGAATCGCTTTCTGG		
*SLC2A4*	Human	F	TTGTGGCTGTGGGTCCCAT	153	15
		R	CTCGTCTTAGAAGAGCTGGA		
*ADIPOQ*	Human	F	GCTGTTCTACTGCTATTAGC	196	6
		R	GATCTCCTTTCTCACCCTTC		
*Pparg*	Mouse	F	ACACCAGTGTGAATTACAGC	79	2
		R	TCTGGGTCAACAGGAGAAATC		
*Slc2a4*	Mouse	F	CAAGCGGGTCTCACTAGATC	176	13
		R	AGACTCAGGCGCTGCAATAA		
*Adipoq*	Mouse	F	CCTGTTCCTCTTAATCCTGC	96	3
		R	CAAGTTCCCTTGGGTGGAG		

* Number of CpG residues within the amplified region.

## Data Availability

Data are available on request from the corresponding author.
